# Recent Anti-KRAS^G12D^ Therapies: A “Possible Impossibility” for Pancreatic Ductal Adenocarcinoma

**DOI:** 10.3390/cancers17040704

**Published:** 2025-02-19

**Authors:** Navid Sobhani, Matteo Pittacolo, Alberto D’Angelo, Giovanni Marchegiani

**Affiliations:** 1 Department of Cancer Biology, The University of Texas MD Anderson Cancer Center, Houston, TX 77030, USA; 2 Department of Surgery, Oncology and Gastroenterology, University of Padova, 35122 Padova, Italy; matteo.pittacolo@aopd.veneto.it; 3 Department of Medicine, Northern General Hospital, Sheffield S5 7AT, UK; alberto.dangelo@nhs.net

**Keywords:** PDAC, *KRAS^G12D^*, targeted therapies, tumor microenvironment

## Abstract

**Background**: Pancreatic ductal adenocarcinoma (PDAC) is an aggressive cancer, able to thrive in a challenging tumor microenvironment. Current standard therapies, including surgery, radiation, chemotherapy, and chemoradiation, have shown a dismal survival prognosis, resulting in less than a year of life in the metastatic setting. **Methods**: The pressing need to find better therapeutic methods brought about the discovery of new targeted therapies against the infamous *KRAS* mutations, the major oncological drivers of PDAC. **Results**: The most common *KRAS* mutation is *KRAS^G12D^*, which causes a conformational change in the protein that constitutively activates downstream signaling pathways driving cancer hallmarks. Novel anti-*KRAS^G12D^* therapies have been developed for solid-organ tumors, including small compounds, pan-RAS inhibitors, protease inhibitors, chimeric T cell receptors, and therapeutic vaccines. **Conclusions**: This comprehensive review summarizes current knowledge on the biology of *KRAS*-driven PDAC, the latest therapeutic options that have been experimentally validated, and developments in ongoing clinical trials.

## Simple Summary

Pancreatic adenocarcinoma is a devastating condition with a dismal prognosis. The *KRAS*^G12D^ mutation is a major driver for this cancer. For many years, it has been considered incurable. The previous generation of therapies were not able to efficiently target the catalytic bonding pocket of the KRAS protein and penetrate this difficult organ. Recently, novel small compounds capable of targeting this region have been discovered, showing promising results. This review describes recent advancements for anti-KRAS^G12D^ agents.

## 1. Introduction

Pancreatic ductal adenocarcinoma (PDAC) is the most common pancreatic cancer, accounting for more than 90% of cases. It is notorious for its poor prognosis, with a 5-year survival rate of less than 10%, largely due to late diagnosis, rapid disease progression, and limited therapeutic options [1,2]. Despite advancements in oncology, PDAC remains resistant to most conventional treatments such as surgery, chemotherapy, and radiation, highlighting the urgent need for more effective, targeted therapeutic strategies.

A pivotal factor in PDAC’s aggressiveness is the high prevalence of mutations in the *KRAS* gene, with over 90% of patients exhibiting activating mutations in this oncogene [3]. Among these mutations, *KRAS*^G12D^ is the most frequently observed, accounting for approximately 40% of all KRAS mutations in PDAC [4]. These mutations lead to the constitutive activation of the KRAS protein, which is integral to cell proliferation, survival, and metabolism, as it persistently drives several key downstream signaling pathways, including RAF-MEK-ERK and PI3K-AKT-mTOR [1,5]. This constant signaling promotes uncontrolled cell division, inhibits apoptosis, and contributes to the hallmark features of cancer, including angiogenesis and immune evasion.

For decades, the KRAS protein was considered “undruggable,” primarily because of its lack of a deep binding pocket and its high affinity for guanosine triphosphate (GTP) [6]. While efforts to target other members of the RAS family, such as HRAS and NRAS, have seen some success, KRAS-targeted therapies lagged significantly behind. In a study by Alberts et al., 48 patients with locally advanced or metastatic PDAC received gemcitabine (1000 mg/m^2^) and ISIS-2503 (6 mg/kg/d), an oligodeoxynucleotide antisense inhibitor of HRAS, with response to treatment monitored with imaging every 1.5 months [7]. The median survival time and 6-month survival rate were 6.6 months and 57.5% (95% CI, 44.9% to 73.5%), respectively. At the same time, Ryan et al. evaluated the therapeutic effects of BMS-214662, a nonsedating benzodiazepine that inhabits farnesylation of the HRAS and KRAS oncogenic proteins, in 14 patients with PDAC [8]. The results showed no objective responses, but one patient received this treatment for more than 3.5 years. In a recent phase I/II study, KRAS- or NRAS-mutant advanced solid tumors, including 19 cases of PDAC, received navitoclax, a BCL-xL inhibitor, combined with trametinib, an MEK inhibitor [9]. Patients with PDAC did not show a partial response.

A breakthrough came with the discovery of KRAS^G12C^ inhibitors, such as sotorasib and adagrasib, which have shown remarkable efficacy in cancers harboring this specific mutation, particularly non–small cell lung cancer [10,11]. These inhibitors covalently bind to the cysteine residue in the KRAS^G12C^ mutant, stabilizing the protein in its inactive Guanosine Diphosphate (GDP)-bound state. Unfortunately, this strategy does not apply to KRAS^G12D^, which lacks the cysteine residue necessary for covalent binding, necessitating the development of alternative therapeutic approaches [12,13].

The development of selective KRAS^G12D^ inhibitors has been a significant focus of recent research, culminating in the identification of MRTX1133. Unlike KRAS^G12C^ inhibitors, MRTX1133 is a non-covalent inhibitor that binds to both the active (GTP-bound) and inactive (GDP-bound) forms of KRAS^G12D^ with high specificity and potency [14,15]. Preclinical models have shown that MRTX1133 can inhibit KRAS^G12D^-driven tumors in vitro and in vivo, particularly in PDAC, where the mutation is prevalent. Early studies demonstrated its ability to shrink tumors and inhibit downstream signaling with minimal off-target effects on wild-type KRAS, making it a highly promising candidate for clinical development [15]. Since then, more small compounds against KRAS mutations have been developed, such as pan-KRAS inhibitor BI-2493, protease inhibitor HRS-4642 targeting KRAS^G12D^, and immunotherapies designed for KRAS^G12D^ PDAC.

While KRAS^G12D^ inhibitors represent a leap forward, PDAC poses additional challenges due to its dense stromal environment and high level of tumor heterogeneity. PDAC is characterized by a fibrotic stroma that impairs drug delivery and fosters an immune-suppressive microenvironment, which reduces the effectiveness of targeted therapies and immunotherapies alike [16,17]. Moreover, the high degree of genetic variability within PDAC tumors means that even successful inhibition of KRAS^G12D^ often leads to adaptive resistance through alternative signaling pathways, such as the activation of PI3K-AKT-mTOR or the induction of epithelial–mesenchymal transition [18,19]. These compensatory mechanisms allow tumors to bypass the KRAS blockade, making combination therapies an important area of focus.

Immunotherapy is also emerging as a potential strategy to overcome some of the limitations of KRAS-targeted treatments. CAR T cell therapy, which involves engineering a patient’s T cells to recognize and attack tumor-specific antigens, has shown early promise in preclinical models of KRAS^G12D^-driven cancers. Additionally, therapeutic vaccines that target mutant KRAS proteins, such as Moderna’s mRNA-5671, are being investigated to elicit an immune response capable of recognizing and eliminating KRAS-mutant tumor cells [10]. While these approaches are still in early stages, they offer a complementary strategy to directly target KRAS and may help overcome some of the challenges posed by the tumor microenvironment and resistance mechanisms.

Despite significant progress in developing KRAS^G12D^-targeted therapies, several obstacles remain. The complexity of the tumor microenvironment, genetic heterogeneity, and the rapid emergence of resistance pathways mean that combination therapies, involving multiple inhibitors or the addition of immune-modulating agents, are likely to be required to achieve long-term therapeutic success [15]. As new inhibitors like MRTX1133 move toward clinical trials, understanding these challenges will be crucial for optimizing treatment regimens and improving outcomes for PDAC patients. This review focuses on KRAS^G12D^ as a strong driver of PDAC, details the different therapeutic approaches targeting this mutation, and summarizes ongoing clinical trials.

## 2. Role of KRAS in Cancer Formation

The worst mutation in terms of prognosis is *KRAS*^G12D^, in ampullary adenocarcinoma [20] and in PDAC [21]. KRAS encodes 21 kDa small GTPases, which cycle between a GTP-bound state (“on”) and a GDP-bound state (“off”). This cycle is mediated by guanine nucleotide exchange factors which activate RAS by aiding the exchange of GDP into GTP. On the other hand, GTPase-activating proteins drive RAS-mediated GTP hydrolysis and hence inactivation. The constitutive activation of Ras results in the persistent stimulation of downstream signaling factors, resulting in the activation of the major hallmarks of cancer [22].

To proliferate, cancer cells must have sufficient energy and biosynthetic building blocks. KRAS promotes glucose uptake by increasing glucose transporter GLUT1, which increases the speed by which glycolytic activity occurs, and lactate is produced [23,24]. Such alteration confers a distinct survival advantage evidenced in oncogenic KRAS-bearing cell lines [23].

Mutant KRAS increases GLUT1 expression and other rate-limiting glycolytic enzyme genes, such as hexokinase 1 and 2, phosphofructokinase-1 (Pfk1), and lactate dehydrogenase A, responsible for converting pyruvate to lactate [19]. KRAS upregulates rate-limiting enzymes for hexosamine, and enzymes involved in the pentose phosphate pathway [19]. These pathways have been deeply involved in cancer progression. Additionally, PDAC has a low vascular density and dense stromal components. These aspects are a challenge to the penetration of PDAC and delivery of therapies, such as antibodies [16].The RAS protein is frequently mutationally activated (HRAS, KRAS, and NRAS) in human cancer. The gene is frequently mutated in the top three cancers associated with mortality in the United States (lung, colorectal, and pancreatic). KRAS is the most commonly mutated RAS isoform, and nearly all PDACs are KRAS dependent [25].

According to the Catalogue of Somatic Mutations in Cancer (COSMIC), which provides access to comprehensive human tumor mutation databases, KRAS mutations were expressed in about 22% of analyzed tumors, versus 8% for NRAS and 3% for HRAS [25]. The RAS isoform mutations are codon-specific. About 80% of KRAS mutations occur at codon 12, whereas only a few occur at codon 61. On the contrary, around 60% of NRAS mutations happen at codon 61, in contrast to the 35% encountered at codon 12. HRAS has 40% of its mutations on codon 12 and 40% on codon 61. At the DNA level, KRAS and NRAS have identical sequences encoding Gly12 and Glu61. Additionally, these oncogenic mutations of KRAS, NRAS, and HRAS are in the same amino acid regions, producing equal effects on the encoded protein and its activity [25].

## 3. The *KRAS*^G12D^ Mutation in Solid-Organ Tumors Including Pancreatic Cancer

The *KRAS*^G12D^ mutation appears in 40% of PDAC patients, encoding a GAT sequence, which produces aspartic acid instead of guanine at amino acid position 12 [26]. Among the various KRAS mutations, *KRAS*^G12D^ is the most frequently correlated with worse survival [21,27,28,29,30,31,32,33,34,35,36,37].

The *KRAS*^G21D^ mutation alone has been shown to result in PDAC formation and its protracted onset [38,39]. Additionally, when coupled with a Tp53-inactivating mutation (Tp53^R157H^ [40]), Smad4 mutation [41], or Cdkn2a mutation [39,42], an acceleration in the formation of pancreatic intraepithelial neoplasia can be observed, often rapidly progressing into metastatic PDAC.

In a mouse model of *KRAS*^G12D^-driven PDAC, the loss of the wild-type allele was associated with metastatic disease. Such wild-type allele loss is also observed in human PDAC [43,44]. Interestingly, upon KRAS inactivation in PDAC that is KRAS^G12D^-driven, rapid regression of primary and metastatic tumor growth can be observed in the presence of Tp53 deficiency [19,45]. This shows how KRAS inhibition in p53-deficient PDAC mice could be a useful therapeutic strategy, as will be discussed in the next chapter. Additionally, *KRAS*^G12D^-driven pancreatic tumor formation was significantly impaired in Rac1-deficient mice [46], Pak1-deficient mice or those treated with anti-Pak1 [47], Rala/Ralb-deficient mice [48], Pik3ca variant–bearing mice [49,50], in mice deficient in both Mek1 and Mek2 or mice deficient in both Erk1 and Erk2 [51], and Raf1/Craf-deficient mice [48,51,52].

Ras mutation can be caused by many genotoxic agents. One of the most frequent carcinogenic chemicals is methyl nitrosourea, which targets the second base on the 12th codon of *HRAS* and *KRAS*, generating a G12D mutation in many cancer types [53,54]. In comparison, UV radiation is known to target pyrimidine dimers, mutating *RAS^Q61^* [55]. In lung cancer, two distinctive G12C mutations in *KRAS* occur, namely GC into TA. These G12C mutations are associated with in vitro bulky DNA formation driven by tobacco smoke products [56]. In smokers, this mutation is very common [57]. In pancreatic and colorectal cancers, this mutation is far less abundant [58,59,60]. In colorectal cancer, the codon 13 mutation of KRAS is very common. In advanced colorectal cancer, patients with the G13D mutation did not show a response to cetuximab therapy, an anti–epidermal growth factor receptor [61].

In thyroid carcinoma, significant numbers of mutations of all RAS isoforms have been observed, linked to ionizing radiation and chemical carcinogens [62]. There is a pattern across RAS isoforms. In this type of cancer, 95% of *NRAS* mutations happen at codon 61 and 66% of *KRAS* mutations at codon 12. *HRAS* has 40% of its mutations on codon 12 and 50% on codon 61. Each codon has distinctive mutation patterns, with the KRAS mutation at codon 12 often showing G12D, while HRAS favors G21V [61].

DNA damage and repair following exposure to carcinogens (e.g., benzo[a]pyrene diol epoxide, or BPDE) has also been associated with RAS isoforms. The preferred binding site for BPDE on *KRAS* is on codon 12 [63]. While the amino acid sequence encoded by each isoform is close to identical across many species, the DNA shows significant variation. The exon 1 variation produces different secondary structures, such as hairpin loops and G-quadruplex structures [64]. In acute myeloblastic leukemia, the expression of NRAS is relatively higher [65]. Additionally, some evidence shows that BPDE-driven DNA damage repair at codon 12 of *KRAS* was less efficient than that in *HRAS* and *NRAS* [66]. In short, the 12th codon is both the most targeted by BPDE and is the least repaired across the isoforms. These reasons could potentially explain why KRAS mutations occur so frequently in cancers [25].

KRAS mutation leads to the neoplastic differentiation of the pancreas into PDAC via progression through three grades of precancerous lesions with increasing levels of disorganization and nuclear abnormalities. During this transition, tumor suppressor genes *CDKN2A*, p53, and *SMAD4* are inactivated [67].

The first step leading to PDAC is the progressive differentiation of normal pancreatic ductal epithelium into precancerous lesions, known as pancreatic intraepithelial neoplasia. The *KRAS* gene mutation is an early event initiating over 90% of these low-grade lesions [68]. PDAC cells prefer low levels of intracellular oxygen species (ROS) [69]. Intriguingly, *KRAS*^G12D^ increases the transcription of nuclear factor erythroid 2-related factor (NRF2), which activates the ROS detoxification program, reducing the intracellular environment [70]. Unlike what was initially thought, KRAS-driven PDAC requires low levels of ROS for optimal growth [71,72]. This requirement could be explained by the opposite roles of ROS: while at low levels ROS may promote cell growth, at high levels these species are cytotoxic [73].

In addition, autophagy is required for KRAS-driven growth. The process of autophagy is a highly conserved mechanism that degrades the intracellular components and promotes reprogrammed cell survival when there are metabolic stresses, by providing ATP and building blocks like amino acids, sugars, lipids, and nucleosides [74]. Autophagy generally is controlled by multiple autophagy-related genes (ATGs), as well as multiple signaling components and growth factors [75]. Autophagy can either cause or inhibit tumor formation [76,77]. There is strong evidence suggesting that in RAS-dependent PDAC, autophagy sustains tumor growth [78]. Indeed, high autophagy levels were detected in pancreatic primary tumors and cell lines. Deleting ATG5 and inhibiting autophagy with chloroquine was able to suppress PDAC growth both in cell lines and in animals [79]. On the other hand, the survival of animals with *KRAS*^G12D^-driven tumors was increased using chloroquine. Suppressing autophagy was associated with increased ROS, less mitochondrial oxidative phosphorylation, and higher DNA damage [79]. On the same note, in cell lines including PANC-1, high basal autophagy was needed to sustain growth and survival [80]. These studies concluded that autophagy and mitophagy are required [81] for PDAC to produce bioenergetic intermediates for the TCA cycle and remove damaged mitochondria. Autophagy-driven cell death or senescence was further promoted by oncogenic *KRAS*^V12^ overexpression, further showing that there is a crosstalk between RAS and autophagy based on different cell types and genetics [82,83,84]. Many studies have shown that KRAS mutation in pancreatic cancer is significantly correlated with shorter overall survival, whereas only a few studies have shown an absence of a correlation [30,31,32,33,34,35,36,37]. Recently, a study of 803 patients with PDAC showed that those with *KRAS*^G12R^ had similar overall survival compared to those with wild-type *KRAS* (38 vs. 34 months, respectively), but those with *KRAS*^G12D^ and *KRAS*^Q61^ had shorter overall survival (22 months and 20 months, respectively) [21,85].

## 4. The Distribution of KRAS Mutations in PDAC

**Figure 1:**
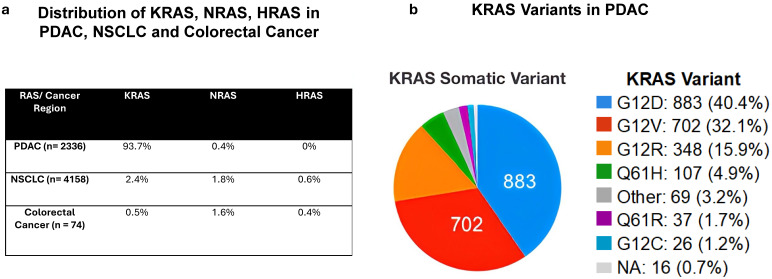
RAS mutational rates. cBioPortal analysis from 2188 pancreatic ductal adenocarcinoma, 4158 non-small cell lung cancer, and 74 colorectal cancer patients. (**a**) The distribution of KRAS, NRAS, and HRAS in these three cancer regions is summarized in the table. (**b**) The distribution of KRAS variants in PDAC is presented in the pie chart.

*KRAS* is the most mutated gene in PDAC (95%), according to several exome sequencing experiments [3,86]. The most common mutation in *KRAS* occurs at the second exon at codon 12 (G12), on the first or second nucleotide, resulting in a conformational change at the site where GTP binds, hence changing the GTP hydrolysis rate [87]. *KRAS* mutations are cancer type-specific, with 98% of PDAC *KRAS* mutations occurring at codon 12, codon 13 (G13), or codon 61 (Q61) [4,5]. These alterations hinder the intrinsic activity of KRAS GTPase activity, blocking the interaction between KRAS and GAPs. Consequently, KRAS becomes constitutively activated and persistently stimulates downstream signaling pathways driving cancer hallmarks [22]. Of the codon 12 mutations, the most frequent are G12D (GAT, 40% of all *KRAS* mutations), G12V (GTT, 33%), and G12R (CGT, 15%), as well as G12C (TGT), G12A (GCT), and G12S (AGT) [85]. Other common mutations at the second exon occur on codon 13, accounting for 7% of KRAS mutations (G13D, G13C, G13S, and G13R); additional mutations are seen at codon 61 of the third exon (Q61H, Q61R, Q61K, and Q61L) and codons 117 and 146 of the fourth exon (K117 and A146) [26]. Further, the sera of patients with KRAS-mutated PDAC contained point mutations in codons 12 and 13 [88]. Moreover, other highly common KRAS mutations in PDAC are *KRAS*^G12V^, which was mutated in 32.5% of cases, and G12R (in 17.1%); less common are G12C (1.7%), G12A/S/L/I (1.4%), G13C/D/P/H/R (1.2%), Q61H (4.8%), Q61K (0.5%), and others (0.5%) [89]. In our analysis of 2188 PDAC patients using cBioPortal data, 93.7% of patients had KRAS mutations, with the following mutations present: G12D (seen in 40.4%), G12V (32.1%), G12R (15.9%), Q61H (4.9%), Q61R (1.7%), G12C (1.2%), other (3.2%) (cBioPortal accessed on 4 September 2024; Figure 1).

## 5. Latest Anti-*KRAS*^G12D^ Therapies

Recently, novel and small molecules have been developed that can target *KRAS*^G12D^ in PDAC.

Sakamoto et al. were among the first groups who discovered selective inhibitory peptides against *KRAS*^G12D^, using random peptide T7 phage display technology. After screening random peptide libraries displaying T7 phage against *KRAS*^G12D^, they subtracted phages bound to wild-type KRAS. They obtained sequences, namely KRpep-2 and KRpep-2d, which bound and selectively inhibited more than 10-fold to *KRAS*^G12D^ than to the wild type, according to SPR and GDP/GTP exchange enzyme assays, respectively. Both KRpep-2 and KRpep-2d had low IC_50_ (8.9 nM and 1.6 nM) and K_D_ (1.6 nM–51 nM), indicating that they were very strong binders and inhibitors [90].

Zheng et al. were also among the first to invent *KRAS*^G12D^ covalently binding inhibitors. The group presented a series of malolactone-based electrophiles, capable of exploiting a ring-strain able to crosslink *KRAS*^G12D^ at the mutated aspartate. They demonstrated that their covalent complexes were strong and stable, exploiting the stereoelectronic requirements for an electrophile attack to be resistant to aqueous solutions. However, they can rapidly crosslink with G12D of *KRAS* in both active (GTP-bound) or inactive (GDP-bound) states. Both in vitro and in vivo data were promising. Their drugs were specific to the mutation in the cell lines and were able to inhibit tumor formation in vivo [91].

Akkapeddi et al. described a series of synthetic monobodies with low nM K_D_ values, selectively binding to *KRAS*^G12D^. These monobodies were designed to target the Switch-II region (S-II) pocket. They were genetically engineered in an expression vector inducible by the presence of doxycycline. Once induced with doxycycline, they inhibited the *KRAS*^G12D^-driven tumorigenic pathway in vitro and tumor progression in vivo [92].

Li et al. used a virtual screening method to discover new thieno [2,3-d]pyrimidine analogs capable of inhibiting *KRAS*^G12D^. Their compounds showed potent antiproliferative activity in the *KRAS*^G12D^ cell lines Panc1, SW1990, and CT26. Their leading compound, KD-8, had an IC_50_ of 2.1 μM and a K_D_ of 33 nM. KD-8 was specific to *KRAS*^G12D^. Furthermore, looking at the mechanisms involved, KD-8 down-regulated the phosphorylation of Erk and Raf in CT26 and SW1990 cancer cells, but not in the wild-type cell line (HeLA). Moreover, in vivo, KD-8 exhibited significant antitumor activity in a CT26 tumor model, without signs of toxicity [93].

Kazi et al. reported the discovery of KRB-456. This compound inhibits *KRAS*^G12D^ as a dynamic allosteric binder to the pocket within the switch-I/II region. Through this mode of interaction, KRB-456 blocks the interaction between *KRAS*^G12D^ and the RAS-binding domain of RAF1. The IC_50_ of KRB-456 was 0.26 µM and the K_D_ was 247 nM. Additionally, the authors showed that KRB-456 binds to *KRAS*^G12D^ with significantly higher affinity than to *KRAS*^G12V^, *KRAS*^G12C^, and *KRAS*^WT^. The authors studied the signaling pathways affected by KRB-456 and observed that the drug inhibited P-MEK, P-AKT, and P-S6 levels in vivo. Additionally, their drug was able to inhibit tumor growth in both subcutaneous and orthotopic xenograft mouse models inoculated with tumors from patients with PDAC *KRAS*^G12D^ and *KRAS*- that had relapsed after chemo- or radiotherapy [94].

Park et al. designed and synthetized pyrimidine and purine analogs with the potential to inhibit the *KRAS*^G12D^ mutation. These compounds had a meaningful anticancer activity in vitro specifically against the *KRAS*^G12D^ mutation. Their leading compound was PU1-1, which exhibited an IC_50_ of 4.4 μM. The mode of action of PU1-1 occurred through the programmed cell death of *KRAS*^G12D^-mutated cells and the reduction in active KRAS. Furthermore, the power of PU1-1 was assessed in an in vivo xenografted PDAC mouse model. The assessment showed that the compound was able to significantly shrink the tumor size, validating its potential therapeutic use [95].

Sun et al. discovered a series of genipin compounds capable of inhibiting *KRAS*^G12D^. Their drugs showed potent antiproliferative effects against *KRAS*^G12D^-mutant tumor cell lines (CT26 and A427). Among their top leading compounds, seven showed micromolar IC_50s_ and were capable of suppressing colon formation of CT26 cells. Their top compound, SK12, significantly induced apoptosis in CT26 cells in a dose-dependent manner. It had a K_D_ value of 157 µM, elevated ROS levels, and modulated the KRAS pathway. Furthermore, the efficacy of SK12 was confirmed in vivo [96].

Unlike *KRAS*^G12C^ inhibitors, selective inhibitors of *KRAS*^G12D^ should bind with high affinity, without a requirement to covalently bind to the mutant KRAS protein. MRTX1133 is the first non-covalent *KRAS*^G12D^ small molecule with a high affinity (K_D_ as low as 0.8 nM) and is a powerful inhibitor, with nanomolar IC_50_ values (5 nM), and has been shown to have efficacy in vivo in xenografted mice [14]. Among the advantages of small inhibitors compared to antibodies are oral delivery, more stability at room temperature, lower cost to manufacture, a large tumor distribution because of their relatively smaller size, and the variety of mechanisms they work through.

The anti-tumor efficacy of the anti-*KRAS*^G12D^ non-covalent drug HRS-4642 with proteasome inhibitor carfilzomib has been demonstrated against the *KRAS*^G12D^-mutant of various solid tumor cancer types both in vitro and in vivo. HRS-4642 had an IC_50_ of 2.329–822.2 nM and a K_D_ of 0.083 nM. Either as a single agent or in combination with carfilzomib, a selective proteasome inhibitor, the drug was able to reshape the tumor microenvironment, making it more immune-permissive and eliciting an immune response against cancer [97].

ERAS-4693 and ERAS-5024 are two oral anti-*KRAS*^G12D^ drugs recently made for solid tumors. In PDAC xenografts, these drugs were given intermittently and showed strong anti-tumor activity. However, there was a strong dose-limiting toxicity, limiting the feasibility of using this drug. More toxicological studies showed that MRGPRX2, linked to pseudo-allergic reactions, was agonized by both ERAS-4693 and ERAS-5024 [98].

The first oral covalent *KRAS*^G12D^-selective inhibitor is RMC-9805. This drug forms a stable, high-affinity novel tri-complex with *KRAS*^G12D^ [99]. It exploits the intracellular chaperone cyclophilin A, forming a non-covalent complex. Overall, the agent forms a binary complex with S-IIP of *KRAS*^G12D^-GTP bound *KRAS*^G12D^, forming a tri-complex of KRAS, cyclophilin-A, and RMC-9805, leading to a covalent G12D cross-linkage, blocking the irreversible downstream binding of KRAS effectors. The interaction causes a selective and persistent modification of *KRAS*^G12D^ by disrupting the downstream *KRAS*^G12D^ signaling effectors (e.g., RAF kinases), thus inducing apoptosis and inhibiting cell proliferation. The authors observed that RMC-9805 was more active in PDAC and non–small cell lung carcinoma than in colorectal cancer models [100].

ASP3082 is the first *KRAS*^G12D^ degrader, which remarkably inhibits *KRAS*^G12D^-mutated cancer models. It is a targeted protein degrader that uses proteolysis-targeting chimera technology to achieve its purpose. The drug entails an E3 ubiquitin ligase-binding moiety conjugated to a *KRAS*^G12D^-binding moiety, via a linker. Once administered, the *KRAS*^G12D^ degrader targets and binds with the *KRAS*^G12D^ moiety of the *KRAS*^G12D^-mutated protein and E3 ligase-binding moiety, forming a ternary complex. This binding induces an E3 ligase-mediated ubiquitination and proteosome-mediated degradation of the KRAS^G12D^-mutated protein. Subsequently, the *KRAS*^G12D^-mediated signaling and activation of the downstream survival pathway is prevented, causing *KRAS*^G12D^-driven tumor repression [101]. ASP3082 showed growth-inhibitory activity in *KRAS*^G12D^-mutated PDAC. Additionally, in vitro, ASP3082 was able to inhibit mutated *KRAS*^G12D^, in contrast with 9000 other proteins, but this was not seen in the wild-type cancer cells. The inhibitory activity of this drug has also been shown in vivo in mice that received an intravenous administration once a week. The effect was dose-dependent and statistically significant, resulting in tumor inhibition without body weight loss, indicating that it could potentially work without toxicity [102].

T cell receptor (TCR)-engineered T cells represent an emerging therapeutic strategy. They are generated by collecting T cells from patients and engineering them in the laboratory to recognize a specific patient’s antigen to be then re-injected in the patients. Autologous *KRAS*^G12D^ HLA-C*08:02-restricted T cell receptor (TCR) gene-engineered T lymphocytes, known as NT-112, target and inhibit *KRAS*^G12D^ with potential antineoplastic activity [103]. The treatment of a PDAC patient with NT-112 showed the successful regression of visceral metastases (overall partial response of 72%, according to the Response Evaluation Criteria in Solid Tumors) for 6 months. Moreover, the study inferred that more than 2% of all the patient’s circulating peripheral-blood T cells had the engineered T cells 6 months after the cell transfer. The overall response rate in the PDAC was mediated in this patient with the help of this TCR gene therapy [104]. Another TCR anti-*KRAS*^G12D^ therapy has been tested in phase I/II clinical trials (NCT03745326). Recently, a study has shown that NT-112 was able to induce tumor clearance in two independent models in vivo. NT-112 T cells were associated with low-frequency chromosomal translocation events (<0.1%) between on-target and off-target Cas9 cleavage sites [105].

**Figure 2:**
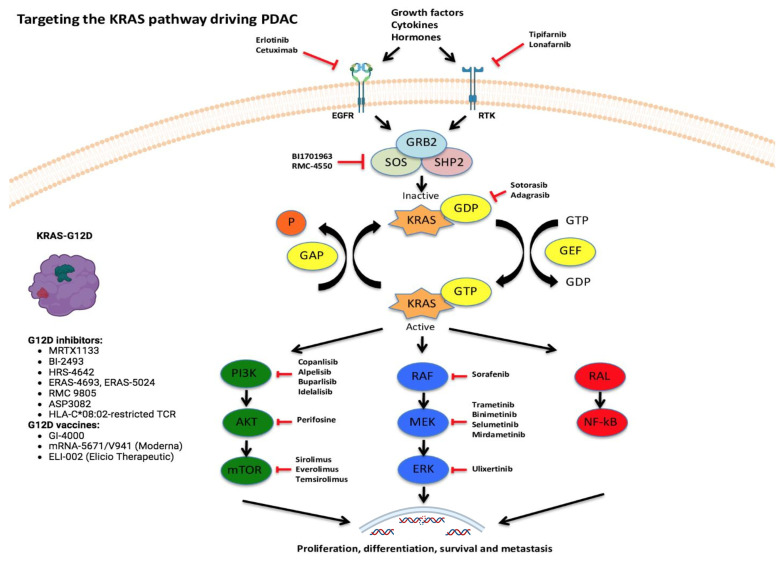
The KRASG12D therapies. The KRAS pathway and therapeutic anti-KRASG12D, vaccination, and CAR-T therapies are considered at the forefront of PDAC research. Abbreviations: EGFR—Epidermal Growth Factor Receptor, RTK—Receptor Tyrosine Kinase, GRB2—Growth Factor Receptor-Bound Protein 2, SOS—Son Of Sevenless, SHP2—Src Homology 2 Containing Protein Tyrosine Phosphatase 2, GDP—Guanosine Diphosphate, GTP—Guanosine Triphosphate, GAP—Guanosine Triphosphate Activating Protein, GEF—Guanine Nucleotide Exchange Factor, P—Phosphorus, KRAS—Kirsten Rat Sarcoma Virus, PI3K—Phosphatidylinositol-3Kinase, AKT—Protein Kinase B, mTOR—Mammalian Target Of Rapamycin, RAF—Rapidly Accelerated Fibrosarcoma, MEK—Mitogen-Activated Protein, ERK—Extracellular Signal-Regulated Kinase, RAL—RAS-Like Proto-Oncogene, NF-kB—Nuclear Factor Kappa B.

A major concern with *KRAS*^G12D^-targeting drugs has been that they can inhibit wild-type KRAS, causing toxicities, even though in preclinical data, such concern has not been observed yet. This makes it crucial for these drugs to be validated in clinical settings, with a small number of patients first, in a dose-escalation manner. Figure 2 summarizes current anti-*KRAS*^G12D^ therapeutic interventional strategies.

## 6. Pan-KRAS Inhibitors in Pancreatic Cancer

The targeting of KRAS is an efficient and promising therapeutic approach for KRAS-driven PDAC, but prevalent KRAS mutation alleles, including G12D, G12V, and G12R, lack targeted compounds [106]. Therefore, pan-KRAS inhibitors could provide a reliable treatment for PDAC with the targeting of all types of KRAS mutations. Wang et al. evaluated the antitumor role of two pan-KRAS inhibitors, BI-2852 and BAY-293, in PDAC [107]. These agents effectively inhibited the proliferation of PDAC cell lines by blocking KRAS activation and can be considered as a promising therapeutic strategy for PDAC. Plangger et al. showed BAY-293, a potent blocker of the interaction between KRAS and SOS1, has synergy with drugs, depending on the tumor type and specific KRAS mutation [108]. In a recent study, ADT-1004, a novel pan-RAS inhibitor, showed inhibition of PDAC growth, an excellent safety profile with profound tumor regression, and no impact on body weight in animal models [109]. Another inhibitor of G12D came from the recent research for pan-KRAS inhibitors, which led to the discovery of BI-2865. The IC_50_ of BI-2865 was 140 nM for the BaF3 pro-B cell line overexpressing *KRAS*^G12C^, *KRAS*^G12D^, and *KRAS*^A146V^. A structural analog of this compound is BI-2493, which was optimized for in vivo administration. The antitumor effect of BI-2493 was associated with the inhibition of phospho-ERK, and DUSP6 expression, in addition to favorable pharmacokinetics [110]. However, additional studies are required to further demonstrate the efficacy of BI-2493 and BI-2865 in different animal models. Table 1 summarizes therapeutic strategies against *KRAS*^G12D^.

## 7. The Role of an Immune-Permissive Tumor Microenvironment

A recent study using spatial genomics of 20 patients and bulk RNA sequencing of 100 tumors has brought to light enhanced epithelial–mesenchymal transition in *KRAS*^G12D^ and increased NKFβ in patients with *KRAS*^G12R^ pancreatic cancer [111]. RMC-9805 in combination with anti-PD-1 therapy showed synergetic activity by shaping a favorable immune microenvironment through cytokines. This reiterates the importance of the tumor immune microenvironment in anti-*KRAS*^G12D^ therapy [100]. The role of the tumor microenvironment in mediating the efficacy of *KRAS*^G12D^ inhibition by MRTX1133 and subsequent resistance mechanisms has been recently investigated using spatial transcriptomics, proteomics, and single-cell RNA sequencing. The drug was associated with higher levels of antigen-presenting cells, T cells, and tumor-restraining fibroblasts close to the cancer cells. These first shreds of evidence are suggestive of a remodeling of the local tumor microenvironment to facilitate the response to the *KRAS*^G12D^ inhibition. The single-cell sequencing data revealed CDK8 as an intrinsic resistance mediator, whereas CXCL2 was an extrinsic resistance mediator to the MRTX1133 drug [112]. The authors showed that antigen-presenting cells (APCs) and CDK8 are potentially useful therapeutic targets that could enable long-term therapy response to the small molecule MRTX1133. With this knowledge in mind, multiple immune cytokines and cell-cycle checkpoint proteins should be further investigated, as they could provide pivotal candidates to be targeted at the same time for optimal *KRAS*^G12D^ inhibition in the PDAC context.

## 8. Combination Strategies

Various combination strategies could potentiate the *KRAS*^G12D^ pathway. *KRAS*^G12D^-inhibitors in combination with various companion inhibitors could provide a more in-depth and durable anti-tumor response. For example, using RMC-9805 with SHP2, mTORC1, or RAS^MULTI^(ON) inhibitors improved the overall response rate up to 60%, delaying the onset of tumor resistance in vivo. In colorectal cancer models, the combination drove regression, where the tumors were usually less responsive to monotherapy. Additionally, RMC-9805 with anti-PD-1 therapy was stronger than the monotherapy in immune-competent mice models with *KRAS*^G12D^ [100]. Farnesyl-transferase (FTase) is one of the three enzymes in the prenyltransferase group, which adds a 15-carbon isoprenoid to the farnesyl group to proteins bearing the CaaX motif. CaaX is a four-amino acid sequence at the carboxyl terminus of a protein. FTase targets RAS-GTP-binding proteins. Inhibitors of FTase are therefore used as anti-cancer agents. Molnar et al. investigated the combination of an FTase inhibitor with an anti-*KRAS*^G12D^ inhibitor. The combination showed a synergistic effect in both 2D and 3D PANC1 PDAC models, which are usually highly resistant to MRTX1133 in 2D models. In addition, the combined therapy was associated with the inhibition of FTase-regulated proteins HRAS and RHEB as well as the expression levels of KRAS [113]. The combination of anti-*KRAS*^G12D^ and MAPK inhibitors with anti-HER2 drug conjugates was more promising than single agents in pre-clinical models. Bulle et al. observed that MAPK inhibition showed rapid phospho-activation of HER2 in PDAC, providing grounds for a therapeutic combinatorial approach. MAPK inhibitors cause proteasomal degradation of dual-specificity phosphatase 6 (DUSP6). The HER2 C-terminal domain has a TEY motif, which is also expressed in ERK1/2, facilitating DUSP6 binding. This enhances the phosphatase activity and dephosphorylation of HER2. Together with MAPK inhibitors, DUSP6 is displaced and goes through degradation. The combination of ERK and HER inhibitors in PDAC xenografted models slowed tumor growth. However, to achieve tumor regression, anti-HER2 trastuzumab conjugated with a cytotoxic chemotherapy payload had to be used in combination with the MAPK inhibitors. There was no noticible toxicity, showing that this treatment combination could be both efficient and safe [114].

## 9. Ongoing Clinical Developments

Multiple clinical trials are testing the safety and efficacy of *KRAS*^G12D^ therapy in solid tumors, including for PDAC. A phase I trial of HRS-4642 has demonstrated the drug’s safety in 18 patients with solid tumors [115]. RMC-9805 has been tested in early-phase I/IIb clinical trials of *KRAS*^G12D^ solid tumors (NCT06040541), assessing the safety and tolerability of the drug in 290 patients with KRAS-driven solid tumors. ASP3082 has been tested in a dose-escalation phase I study to determine its safety and tolerability in a small group of patients with metastatic solid tumors (3–12 patients). The next expansion study, further evaluating safety and tolerability, may enroll fewer than 20 patients (NCT05382559) [116].

Vaccines directed against *KRAS*^G12D^ are also being studied (Figure 2). The GI-4000 vaccine exhibited a striking effect in a mouse model, reducing the tumor burden by >80% compared to the adjuvant alone. The vaccines elicited a strong immune response in these animals, resulting in the secretion of Th1 cytokines. However, the release of Th2-related cytokines was minimal [117]. A phase II clinical trial testing GI-4000 against *KRAS*^G12C/D/V^ in lung carcinoma showed that 50% of patients showed an immune response to mutant *KRAS*^G12C/D/V^, and overall survival showed a positive trend [118]. Another KRAS vaccine is mRNA-5671 (Moderna). This tetravalent vaccine is formulated in a lipid nanoparticle that targets four of the most commonly occurring KRAS mutations, G12D, G12V, G13D, and G12C. Once administered, mRNA-5671 is taken up and translated by APCs. After translation, the epitopes are presented by major histocompatibility complex molecules expressed on the APCs. Consequently, both cytotoxic T-lymphocytes and memory T cells are activated against tumor cells harboring these specific KRAS mutations [117,119]. Another *KRAS*^G12D^ vaccine, for PDAC and colorectal cancer, is ELI-002 2P. It enhances lymph node delivery and immune response through the use of amphiphile modifications of G12D and G12R KRAS peptides when given together with CpG oligonucleotide adjuvant. Encouraging immunogenicity and relapse-free survival were observed in 25 patients (20 with PDAC and 5 with colorectal cancer) in a phase I dose-escalation study (NCT04853017). Ongoing clinical trials in solid tumors, including PDAC, are summarized in Table 2.

## 10. Discussion

Among the most lethal malignancies, PDAC is one of the most difficult to treat, and the current standard methods have so far been proven inefficient. Moreover, PDAC is usually diagnosed at late stages, when the tumor has already metastasized to other parts of the body, further limiting the available therapeutic tools.

Given this need, the efficacy of novel gene therapies or targeted therapies is currently being tested in laboratory and clinical settings. Among the advantages of covalent drugs is that, even at low concentrations, they can bind strongly to their targets because of their picomolar IC_50_ concentrations; and they are more selective, lowering the risk of adverse effects; moreover, their duration of action is longer. Among the disadvantages are off-target toxicity and the risk of having excess immunogenicity [120]. Non-covalent drugs could address some of these important issues, providing that their dose of administration is fine-tuned to obtain reasonable therapeutic indexes. New non-covalent drugs have been tested. In addition to the MRTX1133 *KRAS*^G12D^ inhibitor, BI-2865 is a pan-KRAS inhibitor that also binds to GDP-associated wild-type and mutant *KRAS* with high affinity. The latter drug was later optimized into BI-2493 for in vivo use [121]. However, their therapeutic efficacy should be reiterated in more genetically modified animal models and further explored in both animal and clinical trials. Additionally, in cell lines and organoids, resistance to the anti-*KRAS*^G12D^ MRTX1133 was shown to be mediated by the PI3K-AKT-mTOR pathway and epithelial–mesenchymal transition proteins [122]. It appears that co-evolution of resistance to anti-KRAS^G12D^ therapies exists in the human body. Therefore, multiple combinational therapies, including the modulation of a favorable immune microenvironment, could be the more appropriate approach to PDAC.

Despite significant progress in therapies targeting *KRAS*^G12D^, several limitations persist. A key challenge is the intrinsic heterogeneity of KRAS mutations and the complex genetic landscape of PDAC beyond KRAS itself. Various mutations and emerging variants complicate the development of universal and effective therapies. Additionally, concomitant mutations in genes such as p53, *SMAD4*, and *CDKN2A* can influence therapeutic responses, necessitating combinatorial and personalized approaches [9,62,63].

Although *KRAS*^G12D^ is the predominant mutation, other variants, such as *KRAS*^G12C^ and *KRAS*^G13D^, further complicate the development of inhibitors with broad efficacy. *KRAS*^G12C^ inhibitors have shown promise in certain cancers, but their efficacy in PDAC remains unclear due to genetic variability and the complex tumor microenvironment. Several studies still underscore the challenges of targeting KRAS mutations. *KRAS*^G12C^ inhibitors like sotorasib and adagrasib have demonstrated potential, particularly in non–small cell lung cancer, but their success in PDAC is less definitive due to the tumor’s genetic heterogeneity and challenging microenvironment. *KRAS*^G12C^ is also known to promote a pro-inflammatory tumor microenvironment, which may undermine the long-term efficacy of these inhibitors in solid tumors like PDAC. Furthermore, KRAS variants—including *KRAS*^G12D^, *KRAS*^G12C^, and *KRAS*^G13D^—exhibit different biochemical properties, such as varying rates of GTP hydrolysis and interactions with downstream effectors. This variability complicates the development of inhibitors that can effectively target multiple KRAS variants across diverse tumor types [122,123,124]. A promising KRAS^G12V^ inhibitor is JAB-23000. This compound binds to KRAS^G12V^ in the GTP-active state [125]. Pan-KRAS inhibitors are an interesting strategy, capable of targeting multiple RAS with one drug. ADT-007 is a pan-Ras inhibitor, with a potent anticancer activity. This compound inhibited the activation of KRAS GTP and prevented MAPK/AKT signaling. It binds to the RAS.ADT-007 nucleotide region. The effect of this drug results into mitotic arrest and apoptosis. It was efficacious in both syngeneic immune-competent mice and xenogeneic immune-deficient mice for CRC and PDAC [126]. Based on this discovery, ADT-1004 was developed, its bioavailable prodrug version [109]. Beyond targeting specific KRAS subtypes, there is an urgent need for clinical trials to validate the efficacy and safety of these novel drugs in larger populations and more complex preclinical models [87,88]. Alarmingly, resistance models are emerging, with some studies highlighting the activation of alternative pathways, such as PI3K-AKT-mTOR and epithelial–mesenchymal transition-related proteins, which may counteract the inhibitory effects of anti-*KRAS*^G12D^ therapies [127]. Developing new therapies against PDAC has been challenging, because of the scarcity in animal models recapitulating this specific type of tumor. The intrinsic internal location of the pancreas requires very specialized technical skills to be able to generate orthotopic tumors in the laboratory. The subcutaneous injection is a good model, but it is limited as it does not fully recapitulate the pancreas function, which is a very complicated organ, whose function is crucial for the survival of the organism. Consequently, these problematics have always been a limiting factor to many research groups in orthotopic testing of their anti-PDAC effects in animal models. This has been limiting the rate at which new drugs have been discovered for this condition.

As technology advances, faster ways of making new compounds are evolving that could best target KRAS. The first and more recent generations of translational targeted anti-KRAS therapies hold the promise of improving PDAC.

## 11. Conclusions

Currently, there are multiple experimental analyses focused on KRAS-derived targets, but they have not been incorporated in the clinical setting as much as perhaps they should be. For a long time, treating KRAS was considered to be impossible, mainly because it was very difficult to find a drug that could bind to it with high sensitivity to the complicated chemical structure of this protein. The high affinity for GTP/GDP made this task much harder. However, with the recent advancements in new small molecules that can reversibly or irreversibly bind to KRAS with high affinity, we have a possible impossibility for the treatment of cancers highly reliant on KRAS, such as PDAC.

## Figures and Tables

**Table 1:** Latest discovered therapies for targeting of KRAS^G12D^.

Molecule Inhibitor	Therapeutic Strategy	Covalent or Non-Covalent	Binding Pocket	Interaction with GDP OFF State vs. GTP ON State	IC_50_
KRpep-2 and KRpep-2d	Inhibitor peptide of KRASG12D-SOS1 interaction.	Covalent	Binding peptide sequence: KRpep-1 (Ac-PPWYMCYPMKLKPDC-OH), −2 (Ac-RRCPLYISYDPVCRR-NH_2_), and −3 (Ac-CMWWREICPVWW-OH)	GDP OFF state	8.9 nM and 1.6 nM
Malolactone-based electrophiles	Inhibitor KRASG12D compound decreasing binding KRASG12D to RAF-RBD.	Covalent	A ring-strain able to crosslink KRAS^G12D^ at the mutated aspartate	Binds to either state	GI50 < 10 uM
Monobodies	Monobody genetically engineered in an inducible expression vector by presence of doxycycline. It degraded KRASG12D protein.	Covalent	Switch-II region (S-II) pocket	Binds to either state. A stronger binding to the GTP-ON state.	54 nM
KD-8, a Thieno [2,3-d] pyrimidine analog	Inhibitor KRASG12D compound decreasing levels of pRaf and pErk, in a dose-dependent manner.	Covalent	S-II pocket	Binds to the GTP-ON state	2.1 μM
KRB-456	Inhibitor KRASG12D compound. It is a dynamic allosteric blocker of binding pocket and inhibits binding of KRASG12D to RAF1.	Covalent	SI-II pockets	Binds to the GTP-ON state	0.26 µM
PU1-1, a synthetized pyrimidine and purine analog	Inhibitory compound of KRASG12D. It decreased pERK, pAKT, and pS6 levels in dose-dependent manner.	Covalent	S-II pocket	Binds to the GTP-ON state	4.4 μM
SK12	Inhibitory Genipin compound of KRASG12D, which significantly reduced expression levels of phosphorylated ERK and phosphorylated AKT.	Covalent	Catalytic active pocket of KRAS^G12D^	Binds to either state	Low-micromolar-range IC_50s_
MRTX1133	Inhibitory compound of KRASG12D, which prevents SOS1-catalyzed nucleotide exchange and/or formation of KRAS G12D/GTP/RAF1 complex.	Non-covalent	S-II pocket	Binds preferentially to the GDP OFF state.	5 nM
HRS-4642	Inhibitory compound selectively inhibited KRASG12D, preventing KRASG12D, SOS1, and RAF1 binding.	Non-covalent	S-II pocket	Binds to either state	0.72–822.2 nM
ERAS-4693 and ERAS-5024	Inhibitory KRASG12D compounds.	Covalent	S-II pocket	Binds to either state	0.2 uM M- > 30 uM
RMC-9805	Inhibitory KRASG12D compound. It makes tri-complex with KRAS, cyclophilin-A, and RMC-9805, blocking RAS signaling.	Covalent	S-II pocket	Binds to GTP ON State	EC50 of 17–23 nM
ASP3082	First degrader compound anti-KRAS G12D. It tags protein for proteosome degradation.	Covalent	S-II pocket	Binds to either state	-
NT-112	This CAR-T features HLA-C*08:02-restricted T cell receptor (TCR) gene-engineered T lymphocytes targeted against KRASG12D.	Non-covalent	TCR	TCR	TCR
BI-2852	Pan-KRAS inhibitor compound to WT, G12C, G12D, G12V, and G13D. It blocks GEF, GAP, and KRAS effectors’ interactions and downstream signaling/proliferative pathway.	Non-covalent	SI/II-pocket	GDP OFF State	490–770 nM
BAY-293	This pan-KRAS-SOS1 inhibitor compound blocks RAS activation via disruption of the KRAS-SOS1 interaction.	Covalent	Hydrophobic pocket (Phe-out) and His-Tyr sandwich	GDP OFF State	21 nM
ADT-1004	This is pan-RAS inhibitor compound against G12D, G12V, G12C, or G13Q.	Covalent	Binds to RAS in a nucleotide-free conformation	GDP OFF State	2 nM
BI-2865 and BI-2493	This pan-RAS inhibitor compound is specific to WT, G12C, G12D, G12V, and G13D, and inhibits phospho-ERK, and DUSP6 expression.	Non-covalent	Binds to a conserved binding pocket that partly overlaps with the binding site for covalent inhibitors	GDP OFF State	140 nM

**Table 2:** Ongoing clinical trials testing anti-KRAS^G12D^ therapies in solid tumors.

Trial Identifier	Investigation Plan	Viral Vaccine/ Drug	Clinical Setting	Primary Endpoint	Phase	Trial Status
NCT06385925	Non-randomized, sequential assignment, open label	TSN1611	First line	DLT	I/II	Recruiting
NCT06385678	Non-randomized, single-group assignment, open label	HRS-4642; adebrelimab; SHR-A1921; chemotherapy: pemetrexed, cisplatin, carboplatin	First line	DLT, RP2D, ORR	Ib/II	Recruiting
NCT06500676	Single-group assignment, open label	GFH375	First line	DLT, ORR	I/II	Recruiting
NCT05737706	Non-randomized, sequential assignment, open label	MRTX1133	Second or later line	DLT, ORR, DOR, PFS, OS	I/II	Recruiting
NCT06478251	Non-randomized, parallel assignment, open label	NW-301 TCR-T, NW-301D TCR-T	First line	DLT	I	Recruiting
NCT06403735	Non-randomized, single-group assignment, open label	QLC1101	First line	DLT	I	Recruiting
NCT03745326	Non-randomized, sequential assignment, open label	Drug: cyclophosphamide Drug: fludarabine Drug: aldesleukin Biological: anti-KRAS G12D mTCR peripheral blood lymphocytes	First line	DLT; PR + CR	I/II	Recruiting
NCT06218914	Sequential assignment, open label	NT-112	First line	DLT	I	Recruiting
NCT05533463	Non-randomized, single-group assignment	HRS-4642	First line	DLT	I	Recruiting
NCT06364696	Non-randomized, sequential assignment, open label	ASP4396	First or later line	DLT, ORR, DOR, DCR, PFS, OS	I	Recruiting
NCT06040541	Non-randomized, parallel assignment, open label	RMC-9805 RMC-6236	Second line	AEs, DLT	I/Ib	Recruiting
NCT05382559	Non-randomized, sequential assignment, open label	ASP3082; cetuximab; chemotherapies	First line	AEs, DLT	I	Recruiting
NCT06546150	Single assignment, open label	RE002 T cell	First line	AEs	I	Not yet recruiting
NCT05254184	Single assignment, open label	Mutant KRAS-targeted long peptide vaccine	First line	AEs	I	Recruiting
NCT06484790	Non-randomized, parallel assignment, open label	NW-301V; NW-301D	First line	DLT	I	Recruiting
NCT06484556	Non-randomized, parallel assignment, open label	NW-301V; NW-301D	First line	DLT	I	Recruiting
NCT06487377	Single assignment, 3 + 3 dose escalation, open label	IX001 TCR-T	First or Later line	AEs, DLT	I	Recruiting
NCT06428500	Non-randomized, sequential assignment, open label	QTX3046	First or Later line	TEAEs, DLTs	I	Recruiting
NCT06520488	Single-group assignment, open label	HRS-4642	First or Later line	MTD	Ib/II	Not yet Recruiting
NCT06227377	Non-randomized, single-group assignment, open label	QTX3034	First line	DLT, TEAEs	I	Recruiting
NCT05726864	Randomized, sequential assignment, open label	ELI-002 7P	First or Later line	AEs	Ia, Ib, II	Recruiting
NCT05786924	Non-randomized, sequential assignment, open label	BDTX-4933	First line	MTD	I	Recruiting
NCT06447662	Non-randomized, sequential assignment, open label	PF-07934040	First or later line	AEs, DLT	I/IIa and IIb	Recruiting
NCT06179160	Non-randomized, sequential assignment, open label	INCB161734; cetuximab; retifanlimab	First line	DLTs, TEAEs	I	Recruiting
NCT05846516	Non-randomized, sequential assignment, open label	VSV-GP154; ATP150; ATP152; ezabenlimab	First line	DLT, DFS	Ib	Recruiting
NCT05983159	Non-randomized, parallel assignment, open label	Alpelisib; mirdametinib	First line	VM-PSOM	II	Not yet Recruiting
NCT06208124	Treatment, single-group assignment, open label	IMM-6-415	First or later lines	DLT	I/IIa	Recruiting
NCT05585320	Non-randomized, parallel assignment, open label	IMM-1-104 monotherapy (treatment group A); IMM-1-104 + modified gemcitabine/nab-paclitaxel (treatment group B); IMM-1-104 + modified FOLFIRINOX (treatment group C)	First line or later line	AEs, DLTs, RP2D, OS	I/IIa	Recruiting

## Data Availability

The data supporting the review can be found in the cited primary research articles.

## Abbreviations

AE, adverse event; DCR, disease control rate; DFS, disease-free survival; DLT, dose-limiting toxicity; DOR, duration of response; MTD, maximum tolerated dose; ORR, overall response rate; OS, overall survival; PR + CR, partial response and complete response; RP2D, recommended phase II dose; TEAE, treatment-emergent adverse event.
